# Psychiatric Morbidity, Perceived Stress and Ways of Coping Among Parents of Children With Intellectual Disability in Lahore, Pakistan

**DOI:** 10.7759/cureus.2200

**Published:** 2018-02-16

**Authors:** Muhammad H Sheikh, Sania Ashraf, Nazish Imran, Sadia Hussain, Muhammad W Azeem

**Affiliations:** 1 Psychiatry, King Edward Medical University Lahore, Pakistan; 2 Child and Family Psychiatry Department, King Edward Medical University Lahore, Pakistan; 3 Psychiatry, King Edward Medical University/Mayo Hospital, Lahore, Pakistan; 4 Psychiatry, Sidra Medicine

**Keywords:** intellectual disability, depression, coping, children, pakistan

## Abstract

Background

This study assessed anxiety and depression levels among parents of children with intellectual disability (ID) and analyzed their coping strategies.

Methods

One hundred parents of children with ID were recruited through child psychiatry outpatient services in a tertiary care setting in Lahore, Pakistan. A structured questionnaire including sociodemographic details, the Agha Khan University Anxiety Depression Scale, the Family Stress and Coping Questionnaire, Brief COPE questionnaire, and Support questionnaire were used for data collection.

Results

The mean age of parents was 35 years, and the majority of parents (86%) in the study were females. Seventy percent of the parents had significant levels of anxiety and depression. Parents mostly used emotion-based coping to deal with their anxiety and depression; self-distraction, behavioral disengagement, and venting were the main coping strategies used.

Conclusions

The study showed family stressors, various coping strategies, and support sources in depressed parents who are taking care of their intellectually disabled children. Based on these results, effective culturally sensitive intervention programs can be designed to educate parents and help them effectively cope with stress.

## Introduction

Intellectual disability (ID), formerly known as mental retardation [1], is characterized by significant limitations both in intellectual functioning (i.e., reasoning, learning, and problem-solving) and adaptive behavior, which covers a range of everyday social and practical skills. Studies have shown significant psychological distress, particularly anxiety and depression, in parents having children with intellectual developmental disorders [2-3]. Various reasons of perceived stress among such families include greater financial burden, frequent disruption of family routine and leisure [4], a lower sense of coherence, difficulties in communication with healthcare providers, changes in family relationships, level of support within the community, and difficulties in interactions with the school [5]. The level of perceived stress varies with parental age and race. As this stress increases, the quality of life decreases [6].

Parents develop different positive and/or negative coping strategies to combat this psychological stress [7]. Significant differences in coping behavior have been reported among parents with different marital and socioeconomic status [8]. These coping methods also predict the outcomes of their distress, which may increase or decrease depending on the ways of coping [9]. In order to help themselves, parents rely on different sources of support. Strengthening these sources, formal or informal, can help to create a positive impact on their well-being [10].

Although some cross-sectional studies are available from Pakistan looking at the psychological impact of caregiving among parents of children with developmental disorders [3,11], research addressing the parental coping style and presence of social support is scarce. Our study aimed to determine the anxiety and depression levels in parents of children with ID and correlate it with their coping strategies alongside trying to understand their perception of support available to them in a local context. By recognizing the coping strategies used by the parents, mental health professionals can develop effective intervention programs to help these parents overcome their anxiety and depression and find the right ways to support them.

## Materials and methods

We conducted this cross-sectional study at a tertiary care hospital in a lower middle-income country. After approval from our Institutional Review Board, 100 parents of children aged two to 16 with a primary diagnosis of ID (interview based on DSM-V) were recruited from the outpatient clinic of Child Psychiatry Department. Children who were 17 or older and parents who were unwilling to participate in the study were excluded. Following written informed consent, data were collected via a self-administered questionnaire.

Sociodemographic information including urban versus rural residence and marital status was obtained.

Agha Khan University Hospital Anxiety and Depression Rating Scale (AKUADS)

Parental anxiety and depressive symptoms were assessed with AKUADS, a 25-item screening instrument in Urdu language, developed indigenously in the primary health care and psychiatric setting of Pakistan for screening depression and anxiety. It incorporates culturally pertinent somatic metaphors of depressive disorder [12]. Of the questionnaire’s 25 items, 13 are psychological, and 12 are somatic questions. At a score of 20, it has a sensitivity of 66%, a specificity of 79%, a positive predictive value of 83 and a negative predictive value of 60. 

The Family Stress and Coping Questionnaire (FSCQ)

FSCQ [13] was used to assess parents’ level of perceived stress in 19 areas of their lives. It consists of 26 self-report items using a four-point Likert scale ranging from 0 (being not stressful) to 3 (extremely stressful), as well as an open-ended question asking the parents to list their top three sources of stress.

The brief COPE

The brief COPE [14] is a 28-item self-report questionnaire used to assess several different coping behaviors and thoughts a person may have in response to a specific situation. It is made up of 14 sub-scales: self-distraction, active coping, denial, substance use, use of emotional support, use of instrumental support, behavioral disengagement, venting, positive reframing, planning, humor, acceptance, religion, and self-blame. Twenty-eight coping behaviors and thoughts (two items for each sub-scale) are rated on frequency of use by the participant with a scale of one (“I haven’t been doing this at all”) to four (“I’ve been doing this a lot”), and are then grouped in to problem-based and emotion-based coping. The brief COPE scale has good internal consistency and test-retest reliability, and concurrent validity has been established. In health psychology, the brief COPE has predicted clinically relevant outcomes across many stressful situations and populations.

Support questionnaire

The Support Questionnaire developed by Tehee et al. [13], identifies how helpful an informal or formal source of support is to a parent of a child with an autism spectrum disorder. It is modified for the present study to fit the unique challenges the Pakistani population faces. The Support Questionnaire has a total of 12 items (five formal sources and seven informal sources). For both sources, participants could choose to rate each source as poor, satisfactory, excellent, or not available. 

The translation and cultural adaptation of instruments is an internationally recognized method [15]. For translation and linguistic validation of the FSCQ, COPE, and Support Questionnaires, panel members were recruited from different professional backgrounds including psychiatrists, psychologists, pediatricians, and family physicians. A back translation method was used. In the first phase, each questionnaire was translated by two members into Urdu (local language) which were then compared, and the most suitably translated and culturally accepted items were compiled. In the second phase, two different bilingual translators translated the Urdu version back to English; the items were then combined and refined to produce a single version in English. Finally, a panel of bilingual experts who were familiar with the socio-cultural context reviewed and compared the two English copies (the original and back-translated). Suggestions put forward by the panel were incorporated into the final Urdu version with few questions being reworded/phrased based on feedback. These steps were carried out to check for conceptual equivalence and clarity. The modified questionnaires were then pilot-tested on a group of 15 parents for their appropriateness and comprehension. Cronbach’s alpha values for scales in this study were AKUADS, 0.90; brief COPE, 0.82; problem-based coping, 0.71; emotion-based coping, 0.78; FSCQ, 0.91; and the Support Questionnaire, 0.56.

Data were analyzed using IBM SPSS Statistics Version 22.0 (Released 2013. IBM SPSS Statistics for Windows, Armonk, NY: IBM Corp). Descriptive statistics were computed for all data. Independent samples t-test was used to determine any difference between anxiety and depression among the parents of children with ID and ID with comorbidities. Anxiety and depression scores were correlated with FSCQ items, and different coping strategies and support sources were correlated through the Spearman correlation coefficient. A P value < 0.05 was considered significant for all.

## Results

The mean age of children in the study was 9.5 ± 3.5 years with the majority being male (61%). Most children had a moderate ID (44%), followed by severe (40%), mild (14%), and profound (2%) ID. In addition to ID, 54% had a comorbid diagnosis (including epilepsy, autism spectrum disorder, oppositional defiant disorder, and attention deficit hyperactivity disorder).

Depression status of parents

The mean age of parents was 35.4 ± 6.7 years with the majority being mothers (86%). Seventy percent of the parents had significant anxiety and depression with an AKUADS score of more than 19. Statistically significant differences regarding depression were observed between the gender of the parents (mean AKUADS score for fathers was 19.64 ± 11.12, while for mothers it was 28.02 ± 13.58, p = 0.031), and employment status of parents (employed participants had a mean score of 24.58 ± 13.40 while unemployed participants had a mean score of 30.12 ± 13.21, p = 0.04). There was a statistically significant difference in the mean AKUADS scores for parents of children belonging to different ID classes as determined by one-way analysis of variance (ANOVA; F (3, 96) = 2.755, p = 0.047). A Tukey post hoc test revealed that the total AKUADS score was significantly lower when the children belonged to mild ID group (18.57 ± 10.86) as compared to when the children belonged to moderate ID group (29.32 ± 13.66, p = 0.045). For different income groups, a statistically significant difference was found, as determined by one-way ANOVA (F(2,97) = 6.707, p = 0.002). The Tukey post hoc test revealed that the total AKUADS score was significantly higher when the income was less than 10,000 rupees (29.53 ± 13.2, p = 0.001) and between 10,000 to 20,000 rupees (27.36 ± 12.46, p = 0.006) compared to when income was more than 20,000 rupees (13.82 ± 12.29). There was also a statistically significant difference (p = 0.010) between the mean AKUADS scores of parents when they were taking care of a boy (29.62 ± 14.12) versus when they were taking care of a girl (22.51 ± 11.42). No significant difference was observed in depression status of parents with respect to age of parents or children, marital status, the presence of comorbidities in children, their schooling status, and residential situation (i.e., urban versus rural).

Use of different coping styles by parents and its correlation with parental depression

**Figure 1:**
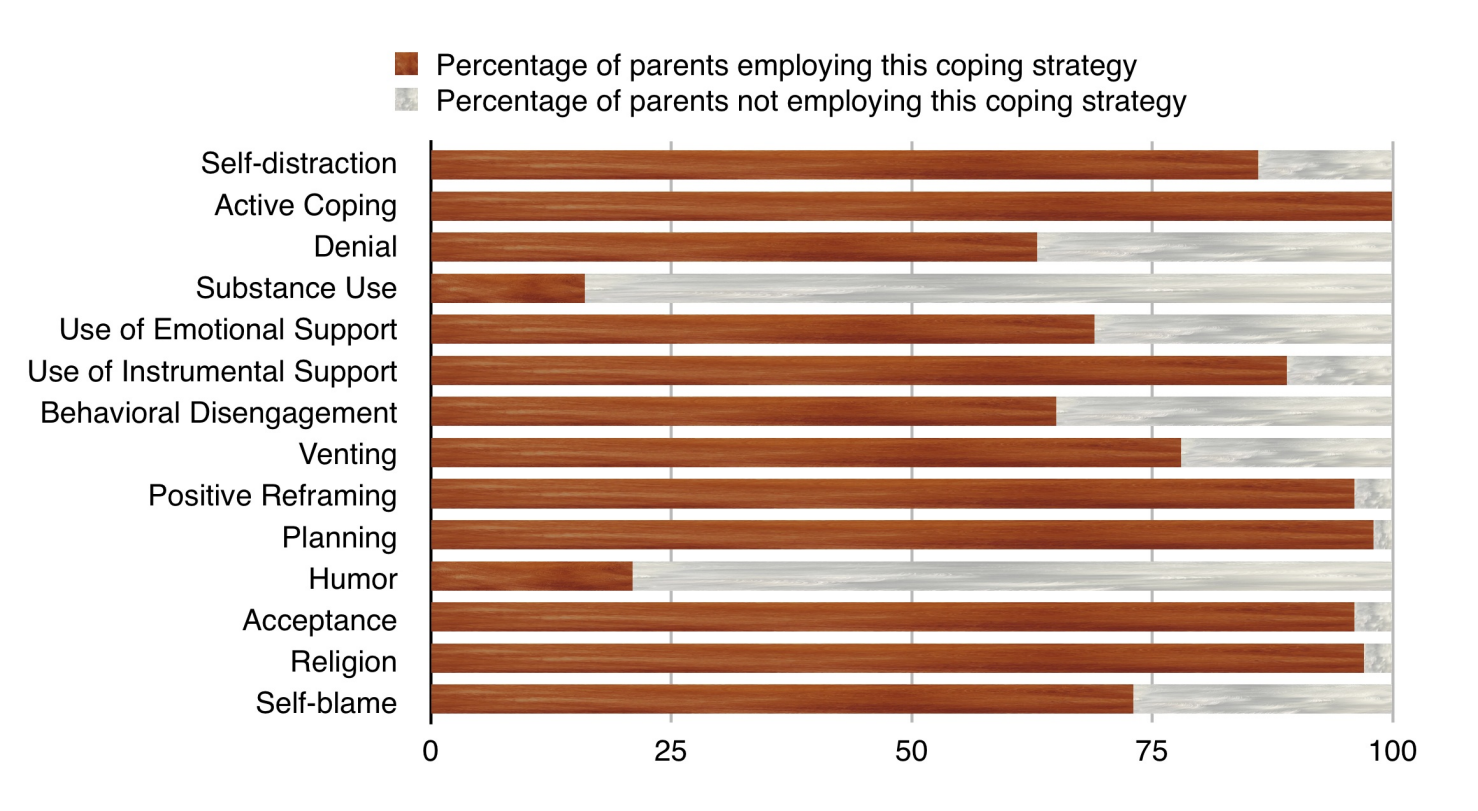
Coping strategies used by parents

The use of different coping styles by parents in our study is shown in Figure 1 with active coping, planning, acceptance, and religion as the more frequently used styles; only 16 % of parents relied on substance use for coping.

The AKUADS score was positively correlated with an emotion-based style of coping. Among the subscales of the brief COPE, the AKUADS score was positively correlated with self-distraction, behavioral disengagement, and venting. The other correlations were not statistically significant (Table 1).

**Table 1:** Correlations of the subscales of brief COPE with total AKUADS score *Correlation significant at p < 0.05 **Correlation significant at p < 0.01 AKUADS - Agha Khan University Hospital Anxiety and Depression Rating Scale.

Brief COPE Sub-Scales	Total Score of AKUADS
Problem-based coping	-.112
Emotion-based coping	.245*
Self-distraction	.266**
Active coping	-.122
Denial	.190
Substance use	.169
Use of emotional support	.029
Use of instrumental support	-.103
Behavioural disengagement	.445**
Venting	.247*
Positive reframing	-.054
Planning	-.052
Humour	.053
Acceptance	-.052
Religion	.101
Self-blame	.150

Parental depression, family stress, and coping

The AKUADS score was positively correlated with most of the FSCQ variables emphasizing that more family stressors are linked to more depressive symptoms. Table 2 shows the correlation of the FSCQ questionnaire variables with the total AKUADS score.

**Table 2:** Correlations of the FSCQ variables with total AKUADS score *Correlation significant at p < 0.05 **Correlation significant at p < 0.01 ID: intellectual disability; AKUADS: Agha Khan University Hospital Anxiety and Depression Rating Scale; FSCQ: Family Stress and Coping Questionnaire.

FSCQ Variables	Total Score of AKUADS
Diagnosis of having ID	.234*
Cause of disability	.237*
Explaining to family	.378**
Explaining to friends	.209*
Explaining to community	.197*
Interacting with family members	.207*
Interacting with friends	.284**
Interacting with community	.211*
Dealing with doctors/other health professionals	.266**
Dealing with therapy providers	.354**
Dealing with teachers	.140
Dealing with the education system	.037
Creating and/or finding opportunities	.342**
Deciding best level of integration	.314**
Meeting needs of other children	.313**
Meeting your own personal needs	.310**
Meeting needs of spouse	.331**
Maintaining personal friendships	.375**
Dealing with your child's sexuality	.381**
Thinking about present/future work placements or employment for your child	.287**
Thinking about present/future long term accommodation for your child	.320**
Planning wills	.441**
Planning emotional and social support	.263**
Planning assistance with care	.175
Attaining respite care	.275**
Dealing with finance issues	.253*

Dealing with financial issues, the child’s inappropriate sexual behaviors, and the children’s integration into the community were the top three stressors reported by parents.

Sources of support identified by parents

**Figure 2:**
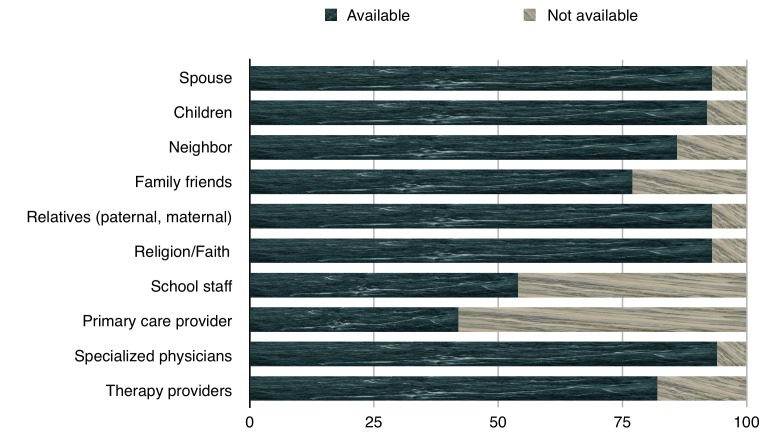
Formal and informal sources of support available to parents

The formal and informal sources of support were available to the parents in different degrees, with more support available generally from informal sources. Spouses, relatives, and religion (93% each) were the top sources of support available from the informal category while primary care provider (58% unavailability) and school staff (46% unavailability) were the least available sources of support from the formal category (Figure 2).

The correlation between the total AKUADS score and different support sources was determined through Spearman correlation coefficient. The AKUADS score was found to be negatively correlated with relatives, specialized physicians, and therapy providers significantly, and emphasized that a lack of support from these resources can contribute to more depressive symptoms (Table 3).

**Table 3:** Correlation between the total AKUADS score and different support sources *Correlation significant at p < 0.05 **Correlation significant at p < 0.01 AKUADS - Agha Khan University Hospital Anxiety and Depression Rating Scale.

Sources of Support	Total Score of AKUADS
Spouse	-.066
Children	-.097
Neighbors	-.164
Family friends	-.134
Relatives	-.274**
Religion/Faith	.010
School staff	.069
Primary care provider	-.013
Specialized physicians	-.199*
Therapy providers	-.209*

## Discussion

Children with ID create a significant impact on the functioning of a family. Multiple studies highlight the evidence of stress and depression among parents [3,16] with their coping behaviors mediating the level of stress in different ways in different socio-cultural settings. The expected risk of psychiatric disorder among the parents of children with cognitive problems can be enhanced by the socioeconomic circumstances of the families [17]. Our findings are consistent with this notion as parents who were employed and earned more than 20,000 rupees per month scored significantly lower on anxiety and depression scale. However, different parental age groups did not differ significantly in anxiety and depression, a result similar to a previous report [18]. This may be because a child with ID represents an ongoing source of loss and grief.

Our finding of mothers being more depressed than fathers is in line with previous reports of mothers being more affected by the need to balance child care needs and household chores, and without additional help, they might feel socially isolated and dissatisfied with life [3,11]. The differences in parenting stress levels can also be because of the different approaches to address the problem, as fathers report more stress related to their child's temperament, whereas mothers report more stress from the personal consequences of parenting [19]. Consistent with the findings by Norlin and Broberg, the stress of meeting the needs of a spouse was also associated with higher anxiety and depression scores in our sample [20].

Family stress tends to increase as the age of child increases. Although with time, the adaptive behaviors and academic skill levels of these children increase, the care demands and behavioral problems of the children with disabilities impose significant stress on parents and contribute to their mental health problems [11]. However, our results contradict this view; the depression and anxiety scores of parents in our sample showed no statistically significant difference based on age groups or schooling status. A poor level of school enrollment and reduced focus on education for children with ID in local settings [21] may be the reason for these findings.

The gender of the child mediates the stress experienced by parents. Compared to some studies highlighting the burden of taking care of daughters with ID to be more stressful [11,22], we found parents of boys had higher depression and anxiety levels. This could be related to the gender norms in the Pakistani culture. Pakistan is a patriarchal society where men are the primary authority figures and women are subordinate. These gender roles determine that men must provide for the families and are supposed to be the family-heads while women take care of the children [23]. Boys also carry the family name, can continue the family trade, and are expected to provide for their parents in old age. Seeing their sons, the ones who would run their families, struggle with mental health can make parents more anxious and more depressed about the future prospects. 

Despite the evidence of the effectiveness of interventions (e.g., behavioral therapy and speech therapy) for children with ID, psychological health issues in parents in our study showed no statistically significant differences in relation to their children's treatment. In the Pakistani setup, the main problem might lie in the lack of rehabilitation facilities, the lack of standard training among staff, and relatively new growth in this field might translate into a lower impact for these kinds of interventions. The fact that renders behavioral therapies less effective in Pakistan as compared to Western cultures can also be attributed to the lack of culturally sensitive adjustments in these therapies. 

Parents listed their child’s inappropriate sexual behaviors and integration in society as their top stressors. It could be due to the therapeutic and ethical dilemmas caused by the psychosexual development of children with ID. In addition, it becomes difficult to integrate the child into the Pakistani community for a variety of reasons. The associated stigma while explaining the condition of their children to family, friends, and the community is one of the more difficult challenges. The stress related to this stigma can stem from multiple sources, for instance, the ‘noticeability’ of the child's speech and behavior, distressing reactions of others, such as staring, displaying discomfort, and inappropriately ignoring or drawing attention to the child [24]. Because of the social stigma towards children with ID, parents feel embarrassed to take their children to social and family gatherings [3]. This can lead to a vicious cycle leading to even lower chances of being part of social programs and gatherings and, hence, hinder the social growth and acceptance of their children.

We observed the emotion-based style of coping in parents to be positively associated with the anxiety and depression scores. Problem-based coping results in reduced stress and an improved quality of the relationship with the child while the use of emotion-based coping negatively correlates with the well-being of parents. Problem-based coping leads to an improved perception of the family towards the child, and this subsequently promotes the mental health of mothers [25]. Anxiety and depression scores were positively associated with venting and behavioral disengagement in our study. Venting emotions and seeking instrumental and emotional support are the kind of coping styles that are adopted more by women [26], and they predict depressive symptoms as do denial and behavioral disengagement [27]. Religious coping and reframing which help parents see the experience of rearing a child with ID in a positive light were used by 97% and 96% of our sample, respectively, highlighting the importance of developing culturally sensitive interventions to improve coping in Pakistan’s context.

In Western cultures, more support is reported from formal sources than from friends or informal sources in contrast with our culture where parents receive minimal support from formal sources. It may be due to the relatively poor organizational structures and less support staff available for providing such support. Grandparents [28] and other close relatives provide substantial support, and parents who receive family support experience less stress. More supportive social networks are associated with better personal well-being and more positive attitudes [29]. Less support from specialized physicians and therapy providers leads to an increase in parent’s anxiety and depression. Studies have highlighted that the most important factor that contributes to the satisfaction of parents with professional support is the adherence to family-centered principles [30]. The lack of these principles might explain the overall dissatisfaction and subsequent increase in anxiety and depression. Hurdles in accessing good services, obtaining relevant information, and ineffective working relationships with professionals can also contribute to negative outcomes. 

## Conclusions

Seventy percent of the parents in our study reported significant levels of anxiety and depression, and they used emotion-based coping most often to deal with their anxiety and depression such as self-distraction, behavioral disengagement, and venting. Our findings help us understand the various methods of coping that parents use to deal with anxiety and depression while raising their children with ID and the availability of different support sources and their role in parental distress. By identifying these coping styles, support sources, and their impact on parental stress, effective intervention programs can be designed to educate parents and to help them effectively cope with stress. Health professionals can mobilize culturally sensitive resources that can be beneficial in a country with limited resources.

Limitations of the study include a small sample size and the inclusion of one study site. The FSCQ and Support Questionnaires used in this study have good internal consistency but are not validated. The majority of parents in this study were mothers. Further studies are needed with multiple sites across the country encompassing both rural and urban settings with a larger sample size. More fathers should be included to understand the impact of parental stress comprehensively.
